# Application of Quantitative Microstructural MR Imaging with Atlas-based Analysis for the Spinal Cord in Cervical Spondylotic Myelopathy

**DOI:** 10.1038/s41598-018-23527-8

**Published:** 2018-03-26

**Authors:** Masaaki Hori, Akifumi Hagiwara, Issei Fukunaga, Ryo Ueda, Kouhei Kamiya, Yuichi Suzuki, Wei Liu, Katsutoshi Murata, Tomohiro Takamura, Nozomi Hamasaki, Ryusuke Irie, Koji Kamagata, Kanako Kunishima Kumamaru, Michimasa Suzuki, Shigeki Aoki

**Affiliations:** 1 Department of Radiology, Juntendo University School of Medicine, Tokyo, Japan; 2 Department of Radiology, Graduate School of Medicine, The University of Tokyo, Tokyo, Japan; 3 Health Science, Tokyo Metropolitan University, Tokyo, Japan; 4 Siemens Shenzhen Magnetic Resonance Ltd, Shenzhen, China; 5 Siemens-healthcare Japan, Tokyo, Japan

## Abstract

Mapping of MR fiber g-ratio, which is the ratio of the diameter of the axon to the diameter of the neuronal fiber, is introduced in this article. We investigated the MR fiber g-ratio, the axon volume fraction (AVF) and the myelin volume fraction (MVF) to evaluate microstructural changes in the spinal cord in patients with cervical spondylotic myelopathy (CSM) *in vivo*, using atlas-based analysis. We used diffusion MRI data acquired with a new simultaneous multi-slice accelerated readout-segmented echo planar imaging sequence for diffusion analysis for AVF calculation and magnetization transfer saturation imaging for MVF calculation. The AVFs of fasciculus gracilis in the affected side spinal cord, fasciculus cuneatus and lateral corticospinal tracts (LSCT) in the affected and unaffected side spinal cord were significantly lower (P = 0.019, 0.001, 0019, 0.000, and 0.002, respectively) than those of normal controls. No difference was found in the MVFs. The fiber g-ratio of LSCT was significantly lower (P = 0.040) in the affected side spinal cords than in the normal controls. The pathological microstructural changes in the spinal cord in patients with CSM, presumably partial axonal degenerations with preserved myelin. This technique has the potential to be a clinical biomarker in patients with CSM *in vivo*.

## Introduction

Cervical spondylotic myelopathy (CSM) is a common spinal cord disorder, especially affecting upper middle-aged people^1^. Disk degeneration, osteophytic changes, and hypertrophy of ligaments result in stenosis of spinal canal, leading to cervical cord myelopathy. The usefulness of conventional MR imaging, including T1- or T2-weighted imaging, is limited to identification of morphological or relative signal changes, providing only non-specific evaluation of the spinal cord. Further, recent advances in neuroprotective approaches^2,3^ may merit early detection of subclinical damage due to CSM, which cannot be detected on conventional MRI. Thus, quantitative MR imaging analysis for assessing spinal cord damage due to CSM needs to be established. In the past, diffusion MRI (dMRI)^4–7^, MR spectroscopy^8^ and other advanced MR techniques and their combinations^9^ have been applied to investigate the spinal cords of patients with CSM, revealing abnormalities in areas with compression. These techniques showed potential to improve the diagnosis and management of various spinal pathologies. However, dMRI is vulnerable to susceptibility artifacts, which is known to be exaggerated in stenosed spinal canal, potentially providing inaccurate metrics^10^. Further, compression of the spinal cord increase the density of neuronal fibers in the spinal cord, which would increase FA^11^. Thus, abnormal quantitative values may or may not be reversible after therapy. Recently, Grabher *et al*.^12^ showed disrupted DTI metrics in the spinal cords of patients with CSM above the level of stenosis. They also revealed correlation of these metrics with clinical disability, suggesting that quantitative MRI in the spinal cord above the level of stenosis could provide unbiased readouts of microstructural damage due to CSM.

Recently, MR fiber g-ratio (the ratio of the diameter of the axon to the diameter of the neuronal fiber) mapping was introduced^13–15^, and it is expected to be a promising tool in the quantitative measurement of the microstructure of neural tissue *in vivo*^16^. Changes in the g-ratio with age and with development in infants have been shown in the literature^17,18^. These reports concern brain tissue, but there is also a report of MR g-ratio mapping *in vivo* in the spinal cord, in which axon density, calculated from the restricted water fraction of dMRI data, and myelin density were measured *in vivo* with high precision and therefore can be used to create a g-ratio-weighted map^19^.

For MR fiber g-ratio mapping, two quantitative MR measurements, the myelin volume fraction (MVF) and the axon volume fraction (AVF), are used to calculate the aggregate g-ratio in a voxel. Several methods of the MVF calculation for quantitative estimation of myelin content have been proposed, such as those based on macromolecular tissue volume^20^, quantitative magnetization transfer (qMT)^21^, magnetization transfer saturation (MTsat)^22^ and relaxometry^23,24^. For AVF calculation to quantitatively estimate axon volumes, diffusion tensor imaging^25^, q-space imaging^26,27^ and neuronal tissue model-based diffusion analysis, such as neurite orientation dispersion and density imaging (NODDI)^28–30^ have been used. These imaging techniques are used for quantitative MR imaging of myelin and axons; therefore, in addition to the g-ratio itself, the myelin and axon structures can be used separately to report the microstructural condition of a spinal cord *in vivo* using these techniques. Moreover, recent developments of the spinal cord atlas with a probabilistic atlas of the spinal cord, e.g., the dorsal column, are available as a part of the Spinal Cord Toolbox (SCT)^31^. Spinal cord analysis based on SCT showed high reproducibility^32,33^ and is expected to describe each anatomic structure in the spinal cord^34^ more precisely than the past arbitrary region-of-interest-based studies.

In this work, we investigate the MR fiber g-ratio, the AVF and the MVF as tools to evaluate microstructural changes in the spinal cord above the level of stenosis in patients with CSM *in vivo* using atlas-based analysis.

## Results

Data from a total of twenty patients and 5 healthy controls were initially included in this study, and data of 4 patients were excluded because their images suffered unexpected artifacts and seemed to be inappropriate for further analysis. Thus, a total of 20 patients with 26 affected sides and 14 unaffected sides and 5 healthy controls with 10 normal sides were analyzed. The demographic and clinical characteristics of the 20 patients and 5 healthy controls are shown in Table 1. The AVF, the MVF and the MR fiber g-ratio values of bilateral DCs and LCSTs are summarized in Table 2. The AVFs of the fasciculus gracilis in the affected side spinal cord and the fasciculus cuneatus and LSCT in the affected and unaffected side spinal cords were significantly lower (P = 0.019, 0.001, 0019, 0.000, and 0.002, respectively) than those of the normal controls.

**Table 1:** Demographic characteristics of the participants.

	CSM patients (n = 20)	Healthy controls (n = 5)	z/X^2^	P
Male/female	9/11	3/2	0.361	0.65
Age (years)	61.1 ± 15.4	45.0 ± 16.1	−1.836	0.71
Affected side				
Left affected	11	0	—	—
Right affected	2	0	—	—
Bilateral affected	7	0	—	—

Values are mean ± SD or n.

X^2^ analysis was used to examine sex and the Mann–Whitney U-test was used to examine age.

**Table 2:**
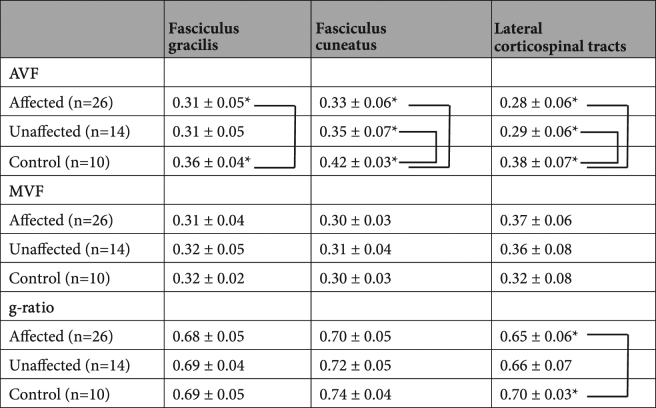
The values represent mean ± SD, a.u. *P < 0.05. Metrics showing significant differences are connected to each other by lines.

**Figure 1:**
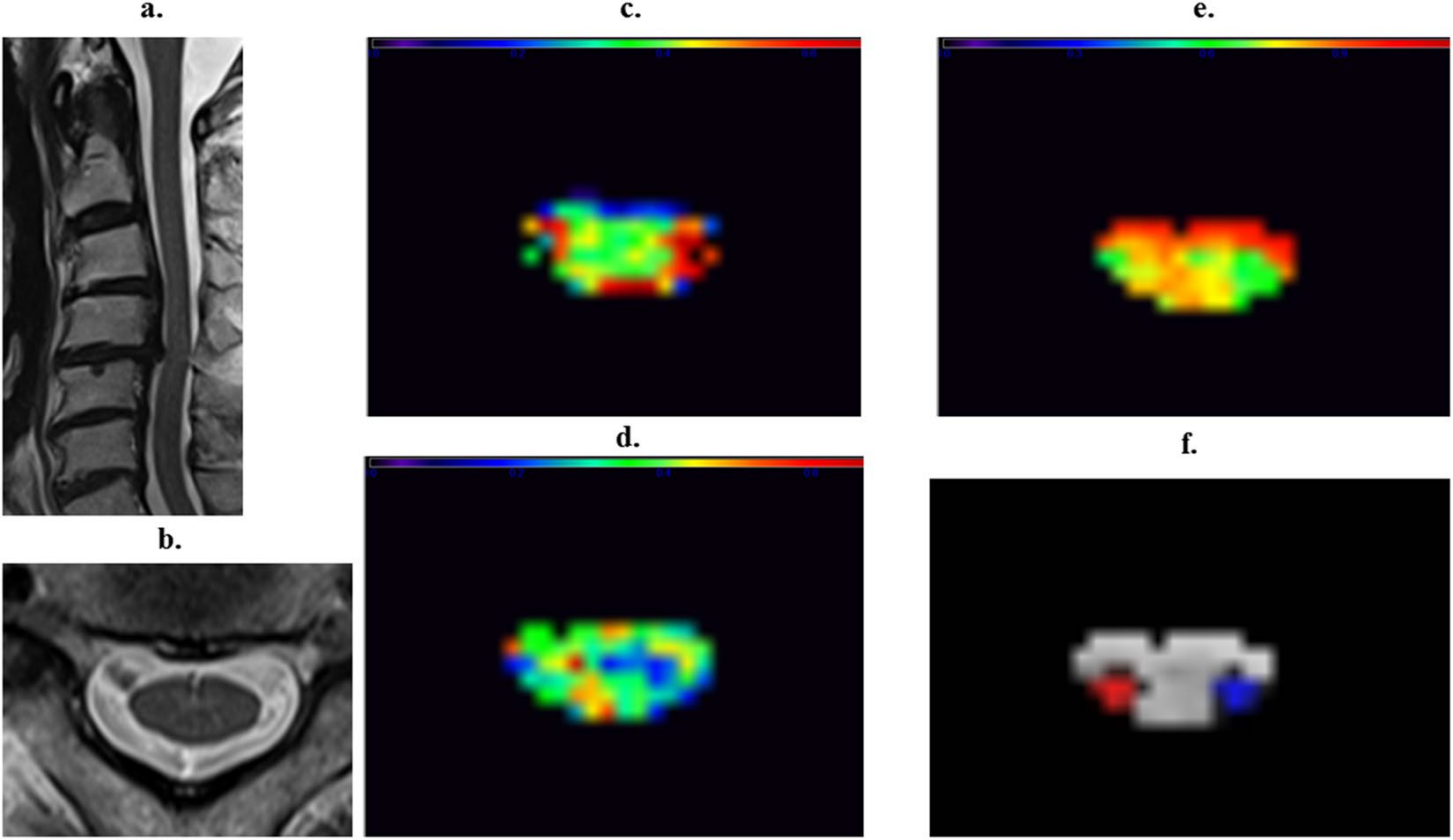
A representative case of cervical spondylotic myelopathy (CSM) (79-year-old male). A sagittal T2-wighted image shows deformation of cervical vertebrae and spinal canal stenosis at C4-5 level (**a**). A transverse T2-weight image at C3 vertebral level shows no abnormal intensity in the spinal cord (**b**). Myelin volume fraction map at C3 vertebral level shows no definite laterality (**c**) but axon volume fraction (**d**) and g-ratio map (**e**) show the lower values in the left lateral funiculi. Right (red) and left (blue) lateral corticospinal tract ROIs of PAM50 atlas co-registered to patient’s space are projected on g-ratio map (**f**). Note that liner high values on the edge of the spinal cord may indicate registration error.

No difference was found in the MVFs. The fiber g-ratio of the LSCT was significantly lower (P = 0.040) in the affected side spinal cords than in the normal controls. A representative case is illustrated in Fig. 1. There was no correlation among mJOA scores, number of levels of compression and the above quantitative MR metrics.

## Discussion

There is very little literature about the g-ratio of normal spinal cord white matter. Duval *et al*. has recently reported that the mean MR g-ratio of a normal spinal cord white matter was 0.76^19^, and past histological studies showed that the normal range of g-ratios in central nervous system was 0.6–0.81^19^. The results of this study showed that the g-ratio values in normal spinal cord white matter were 0.69–0.74, which seem to be reasonable. The normal g-ratio values in our study were lower than that reported by Duval *et al*.^19^, but within the range reported in previous histological studies. The difference can be partly explained by the difference in MRI acquisition sequences for the MVF and the AVF and the calibration methods for MVF estimation.

Our study showed no significant differences in the MVF among 3 groups of spinal cords. A past pathological study showed that there were thin myelinated fibers, indicating focal demyelination and remyelination, in the damaged white matter at the compressed segment^35^. Myelin loss or myelin pallor was not observed in the spinal cords of CSM model rat, even though the disease duration was limited to 25 weeks^36^. In our study, we measured the MVF at the C3 vertebral level, which was above the compressed segment, thus demyelination may not have expanded into the C3 level spinal cord. Another possible explanation is that the MVF may have remained at approximately normal values due to remyelination.

Our results showed that the AVF of the affected side was lower than that of normal controls in all three tracts, with corresponding decrease of g-ratio in LCST. A study of post mortem human spinal cords has suggested that axonal loss and degeneration were observed in the lateral funiculi, including the lateral corticospinal tracts, at the early stage of CSM, whereas the posterior columns were damaged at the later stages^35^. Our AVF results were consistent with these reported pathologic changes. Therefore, measuring the AVF and the g-ratio is an appropriate method to observe the microstructural pathological changes in the spinal cord, which are not demonstrated by conventional MR imaging. Although direct comparison between the disease severity and the MVF, the AVF and the g-ratio was not performed in this study, another study has revealed that MRI shows disease progression more often than clinical measures do, possibly because incremental injury does not necessarily manifest with neurological/functional deficits^37^. Promoting axonal plasticity may be useful to improve neurological function in CSM^38^, so estimation of microstructural changes in the spinal cord by MR imaging including the MVF, the AVF and the g-ratio will play an important role for patient management, with development of regenerative medicine.

There are several limitations in the present study. First, several MR imaging and analysis methods have been introduced for AVF and MVF imaging, and a standard MR imaging method remains to be established. Therefore, our results may be changed by using a different method to calculate the AVF and the MVF. For the AVF, the NODDI model derived from 2-shell dMRI was used in this study, but this was a method generally optimized for brain imaging. A more suitable method for estimating AVF may be possible; for example, double-diffusion-encoded measurements is a good candidate^39^. In addition, even though we used the PAM 50 atlas for localization of each white matter tract, another atlas^40^ may lead to different results.

Another limitation was the small number of patients, and there was no assessment of the correlation between the AVF, the MVF or the g-ratio and patients’ prognosis. Moreover, symptom laterality is not a conventionally classified feature for study as this, although more relevant now in the study using tractography^41^. Longitudinal studies with a larger cohort investigating correlations between clinical outcome and these metrics are needed in the future.

A third limitation was that there was no direct comparison between the AVF, the MVF or the g-ratio and histology. However, Duval *et al*. reported that using histology as ground truth has some limitations, such as degeneration of tissue specimen during preparation for optical imaging^19^. Therefore, at least for clinical use, the correlation between the MR indices and symptoms seems to be more important.

In conclusion, our results show the pathological microstructural changes in the spinal cord in patients with CSM, such as partial axonal degenerations. More studies investigating the correlation between imaging, pathology and clinical assessments are needed. The technique we used has the potential to provide new information and be a *in vivo* clinical biomarker in patients with CSM.

## Materials and Methods

### Subjects

Twenty-four patients with clinically diagnosed cervical spondylotic myelopathy (CSM) (12 women and 12 men, mean age 61 years) and nearly age matched 5 healthy controls without history of neurological disease from local area were enrolled in this study. The mean Japanese Orthopaedic Association score (JOA score) of the patients was 15.6 ± 1.6 (mean ± SD). Symptoms of all the patients were only mild tingling sensation on either or both hands. By referring to conventional MR imaging, including T2- and T1-weighted images, and electronic medical records, including the laterality of symptoms of the patients, two experienced neuroradiologists (R.I. and M.H.) classified the laterality of the disease into two groups, the affected side and the unaffected side. Specifically, affected side was determined if the side of compression of the spinal cord by osteophyte and/or extruded spinal disk confirmed on conventional MR images matched the side of clinical symptom and electronic medical records. Moreover, if a patient had bilateral symptoms and spinal cord is compressed bilaterally, both right and left sides of the spinal cord were considered to be affected. Exclusion criteria for analysis were as follows: (a) the presence of other spinal disease; (b) a history of neck surgery for any disease. In all patients, level of stenosis was at either one or combination of C4/5, C5/6, and C6/7.

### Ethical issues

All data from the patients were obtained in accordance with the 2013 revised Helsinki Declaration of 1964. We provided participants with detailed information, and written informed consent was obtained from all participants. The Ethical Committee of Juntendo University Hospital approved the study.

### Data acquisition

After conventional MR imaging, including T2- and T1-weighted imaging, in the sagittal and transverse planes, quantitative MR imaging data of 2-shell dMRI using a prototype sequence based on simultaneous multi-slice (SMS) accelerated readout-segmented echo planar imaging (rs-EPI), which is essentially multi-shot echo-planar DWI for calculating the AVF and MTsat imaging for calculating the MVF, were acquired with a 3 Tesla scanner (MAGNETOM Skyra, Siemens Healthcare, Erlangen, Germany) with a body coil excitation and 64-ch head/neck coil for reception.

### Axon imaging

We have chosen NODDI for axon estimation in this study because one of the NODDI-derived quantitative metrics, intracellular volume fraction (Vic), is believed to comprise the signal of axons in the white matter^28^. Furthermore, NODDI is more robust for estimating the axon volume in the fiber crossing area than the fractional anisotropy of DTI^15,42^. Thus, NODDI seems to be the best reasonable method available to estimate the AVF in a clinical setting considering its tolerable acquisition time for patients and its reliability. To acquire 2-shell dMRI data for NODDI, rs-EPI sequence was chosen because of its low distortion and relatively high signal-to-noise ratio^43,44^, and accelerated with SMS^45^, which has been shown to cause low level of distortion while preserving the quality of data in the spine^46^.

The imaging parameters for 2-shell dMRI were as follows: repetition time(TR)/echo time, 3000/104 (ms/ms); echo space, 0.50 ms; number of signals acquired, one; section thickness, 4 mm; number of slices, 39; field of view, 200 × 200 mm^2^; matrix, 200 × 200; number of shots, 5; SMS factor, 3; bandwidth, 260 Hz/pixel; parallel imaging using GRAPPA factor 2 in phase-encoding direction; 7/8 partial Fourier acquisition; imaging time, approximately 8 min; 2 b values (1000 and 2000 s/mm^2^) with a b = 0 image and diffusion encoding in 20 directions for each b value.

### Myelin imaging

We have chosen MTsat for myelin estimation in this study because MTsat showed good correlation with qMT^42^, which was thought to be a suitable method for myelin quantification but required a scanning time that was intolerable to patients. In addition, MTsat seems to be a clinically feasible candidate for g-ratio mapping *in vivo*^13^. Three 3D multi-echo fast low-angle shot (FLASH) sequences were performed with predominant T1-, PD-, and MT-weighting. The imaging parameters for MTsat were as follows: MT-off and MT-on scanning (TR/TE = 24/2.53 ms, flip angle = 5 degrees) and T1-weighted imaging (TR/TE = 10/2.53 ms, flip angle = 13 degrees); parallel imaging using GRAPPA factor 2 in phase-encoding direction; 7/8 partial Fourier acquisition in the partition direction; bandwidth, 260 Hz/pixel with the same spatial resolution and slice coverage for 2-shell DWI. Two additional B1 maps using EPI with short acquisition time (about 10 seconds each) were acquired for correction of B1 inhomogeneity using the double angle technique^47^ with 10° and 20° flip angles. Imaging time was approximately 7 min total for MTsat.

### Post-processing and MR fiber g-ratio calculation

MTsat data were analyzed using an in-house MATLAB script for computing the MVF map, based on a theory in the literature^22^. First, apparent longitudinal relaxation rate R_1app_ was calculated as following:

1 $$\,{R}_{1{\rm{app}}}=\frac{1}{2}\frac{{S}_{{\rm{T}}1}{\alpha }_{{\rm{T}}1}/{{\rm{TR}}}_{{\rm{T}}1}-{S}_{{\rm{PD}}}{\alpha }_{{\rm{PD}}}/{{\rm{TR}}}_{{\rm{PD}}}}{{S}_{{\rm{PD}}}/{\alpha }_{{\rm{PD}}}-{S}_{{\rm{T}}1}/{\alpha }_{{\rm{T}}1}}$$

where S_T1_ and S_PD_ denote signal intensities of T_1_-weighted and PD-weighted images, respectively; TR_T1_ and TR_PD_ denote TR of T_1_-weighted and PD-weighted images, respectively; and α_T1_ and α_PD_ denote excitation flip angles of T_1_-weighted and PD-weighted images, respectively.

Second, apparent signal amplitude A_app_ was calculated as following:

2 $$\,{A}_{{\rm{app}}}={S}_{{\rm{PD}}}{S}_{{\rm{T}}1}\frac{{{\rm{TR}}}_{{\rm{PD}}}{\alpha }_{{\rm{T}}1}/{\alpha }_{{\rm{PD}}}-{{\rm{TR}}}_{{\rm{T}}1}{\alpha }_{{\rm{PD}}}/{\alpha }_{{\rm{T}}1}}{{S}_{{\rm{T}}1}{{\rm{TR}}}_{{\rm{PD}}}{\alpha }_{{\rm{T}}1}-{S}_{{\rm{PD}}}{{\rm{TR}}}_{{\rm{T}}1}{\alpha }_{{\rm{PD}}}}$$

Third, apparent MT saturation δ_app_ was calculated as following:

3 $$\,{\delta }_{{\rm{app}}}=({A}_{{\rm{app}}}{\alpha }_{{\rm{MT}}}/{S}_{{\rm{MT}}}-1){R}_{1{\rm{app}}}{{\rm{TR}}}_{{\rm{MT}}}-{{\alpha }_{{\rm{MT}}}}^{2}/2$$

where S_MT_, TR_MT_, and α_MT_ denote signal intensity, TR, and excitation flip angle of MT-weighted image, respectively.

To use δ_app_ as an absolute quantitative marker of myelin, we assumed linear proportional relationship between δ_app_ and MVF^42^. Calibration factor was determined for g-ratio in the corpus callosum to be around 0.7, as suggested by Mohammadi *et al*.^13^. Scanning for this calibration was performed in another cohort of 3 subjects with similar protocol to that used in the current study.

**Figure 2:**
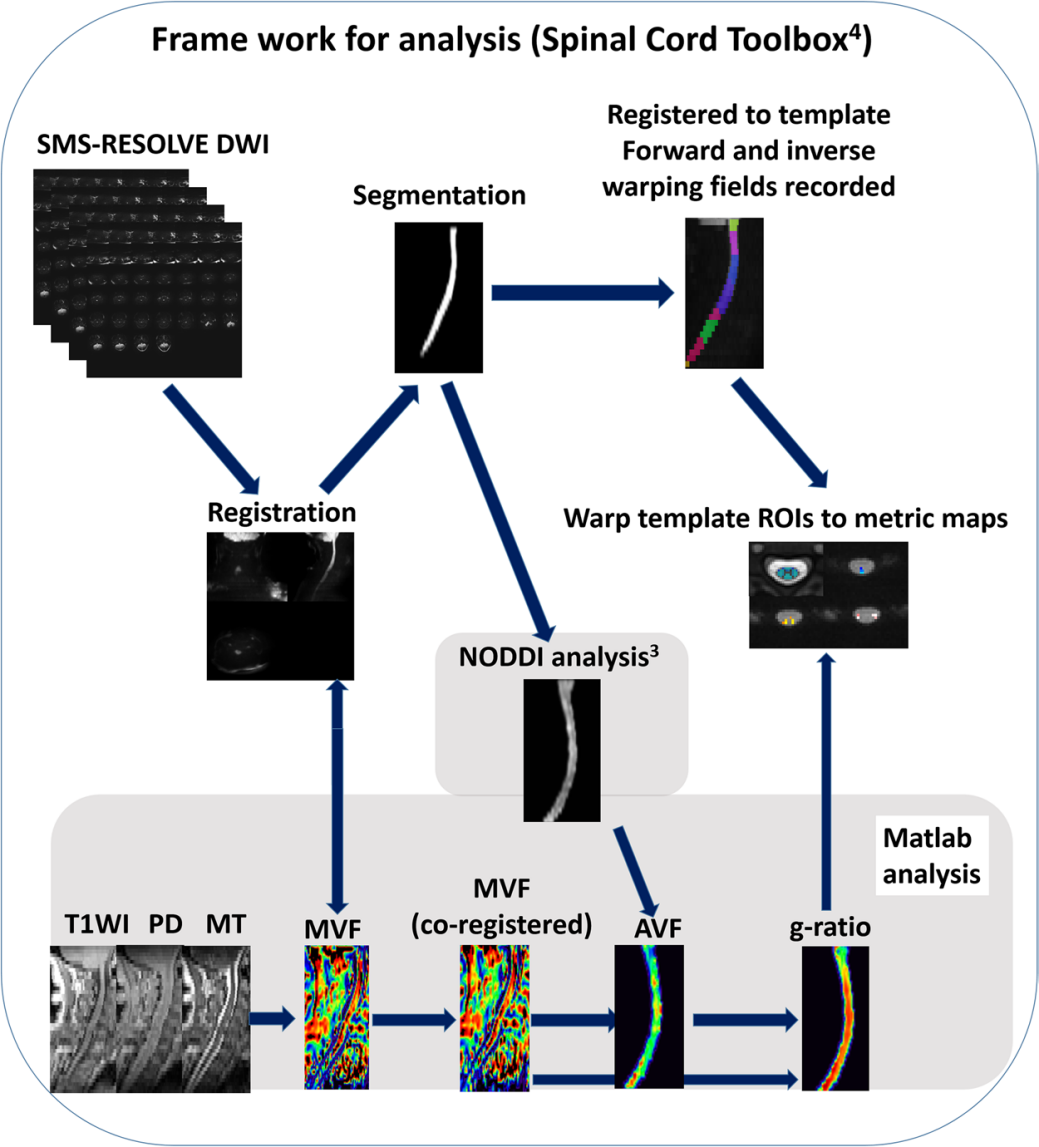
The procedure for generating the myelin volume fraction (MVF), axon volume fraction (AVF) and g-ratio maps. Using the Spinal Cord Toolbox (SCT), diffusion MRI (dMRI) data were registered to each other and segmented in the spinal cord. The spinal cord segmented from magnetization transfer (MT)-weighted image using the SCT was registered to the spinal cord segmented from dMRI data. The MVF map generated from T1-weighted, PD-weighted and MT-weighted images using an in-house MATLAB program was registered to dMRI data using the same warping field. dMRI data of segmented spinal cord were used for NODDI analysis with MATLAB, and the generated ICVF and ISO maps were used for generating the AVF map, followed by the g-ratio map. Segmented spinal cord dMRI data were registered to the PAM 50 template using the SCT, and the template ROI was warped to each subject’s data to measure the AVF, the MVF and the g-ratio in each white matter tract.

For the AVF, neurite orientation dispersion and density imaging (NODDI)^28^ model analysis was employed using dMRI data for Vic, and isotropic water diffusion volume fraction (Viso) indicating free water was calculated using the NODDI MATLAB toolbox (available at https://www.nitrc.org/projects/noddi_toolbox). Then, the AVF was estimated by the following equation: AVF = (1-MVF)(1-Viso)Vic^15^. The aggregate MR fiber g-ratio was calculated as a function of the MVF and the AVF: g-ratio = √ (AVF / (MVF + AVF)). Additionally, semi-automated analysis was performed using the Spinal Cord Toolbox^31^ for segmentation, motion correction, registration to the WM atlas (PAM50 template), co-registration between the MVF and the AVF maps and extraction of metrics. These imaging procedures are summarized in Fig. 2.

In each of the affected and unaffected sides of patients’ spinal cords and spinal cords of healthy volunteers, quantitative metrics in three relatively large white matter tracts in the cervical spinal cord dorsal columns (DCs) at the C3 vertebral level, including the fasciculus gracilis, fasciculus cuneatus, and lateral corticospinal tracts (LCSTs), were selected and compared. To avoid the compressed area of the spinal cord, which could yield biased and incorrect measurements, the C3 vertebral level, which was above the level of stenosis, was selected for ROI analysis^48^.

Moreover, we evaluated among mJOA scores, number of levels of compression and the above quantitative MR metrics.

Statistical evaluations were performed with IBM SPSS Statistics software (version 19.0; SPSS, Chicago, IL), using one-way ANOVA with Scheffé’s post hoc test between the values from affected and unaffected sides of spinal cords in patients and spinal cords in healthy controls. A p-value less than 0.05 was considered to indicate a statistically significant difference.

The datasets generated during and analyzed during the current study are available from the corresponding author on reasonable request.
